# Multimode optical fiber specklegram smart bed sensor array

**DOI:** 10.1117/1.JBO.27.6.067002

**Published:** 2022-06-24

**Authors:** Stephen C. Warren-Smith, Adam D. Kilpatrick, Kabish Wisal, Linh V. Nguyen

**Affiliations:** a University of South Australia, Future Industries Institute, Mawson Lakes, South Australia, Australia; b The University of Adelaide, Institute for Photonics and Advanced Sensing, School of Physical Sciences, Adelaide, South Australia, Australia; c The University of Adelaide, Australian Research Council Centre of Excellence for Nanoscale Biophotonics, Adelaide, South Australia, Australia; d The University of Adelaide, Adelaide Nursing School, Faculty of Health and Medical Sciences, Adelaide, South Australia, Australia; e Royal Adelaide Hospital, Adelaide, South Australia, Australia; f Yale University, Department of Physics, New Haven, Connecticut, United States

**Keywords:** specklegram sensor, multimode optical fiber, optical fiber sensor, sensor array, smart bed

## Abstract

**Significance:**

Monitoring the movement and vital signs of patients in hospitals and other healthcare environments is a significant burden on healthcare staff. Early warning systems using smart bed sensors hold promise to relieve this burden and improve patient outcomes. We propose a scalable and cost-effective optical fiber sensor array that can be embedded into a mattress to detect movement, both sensitively and spatially.

**Aim:**

Proof-of-concept demonstration that a multimode optical fiber (MMF) specklegram sensor array can be used to detect and image movement on a bed.

**Approach:**

Seven MMFs are attached to the upper surface of a mattress such that they cross in a 3×4 array. The specklegram output is monitored using a single laser and single camera and movement on the fibers is monitored by calculating a rolling zero-normalized cross-correlation. A 3×4 image is formed by comparing the signal at each crossing point between two fibers.

**Results:**

The MMF sensor array can detect and image movement on a bed, including getting on and off the bed, rolling on the bed, and breathing.

**Conclusions:**

The sensor array shows a high sensitivity to movement, which can be used for monitoring physiological parameters and patient movement for potential applications in healthcare settings.

## 1 Introduction

Global populations, particularly in developed nations, are aging. For example, up to 10% of Australia’s population is predicted to be above age 85 by the end of the century, up from 1.6% in 2008.^1^ The aging population will add a significant financial and human resources burden on healthcare systems, particularly in the management of chronic conditions.^2^ Future pandemics have the potential to further stretch healthcare resources, as is now well known in the current SARS-CoV-2 pandemic.^3^^,^^4^ Care for patients with chronic illnesses requires laborious monitoring from nurses and other healthcare providers, increasing costs and adding pressure to an already stretched workforce, including the current global shortage of nursing staff.^5^^,^^6^

Nonintrusive sensing technology has the potential to play an important role in managing this challenge. The ability to record vital signs, such as heart rate and respiration rate, as well as patient movement and pressure points, can reduce labor requirements and lead to improved patient outcomes and quality of life.^7^^–^^9^ Sensors exist, particularly wearables, for the detection of many parameters, including physical activity, pressure, temperature, heart rate, respiration rate, blood pressure, blood oxygen saturation, and heart electrical activity.^10^ These are typically point-parameters that are measured at a single location, such as on a person’s wrist. For applications that require spatial information, such as limb movement^11^^–^^14^ or activity on a bed,^15^^–^^17^ an array of either accelerometers or pressure sensors is required. Alternatively, camera-based technology, including depth,^18^ thermal,^19^ and color^20^ cameras, have been widely investigated for bed monitoring for applications such as sleep studies and falls prediction and detection. However, camera-based monitoring can raise issues of privacy and is therefore limited in which application settings it would be tolerated. There exists a need for scalable and cost-effective sensor technology that can output spatial information while being robust, unintrusive, and discrete.

Optical fiber sensors have been implemented in numerous biomedical and biomechanical applications due to their properties of being flexible, lightweight, and surprisingly strong.^21^^–^^23^ A key differentiator from point-wise electronic sensors is that the cable itself can form the sensor, allowing for sensitive accumulated measurements along their length or even spatially resolved measurements using specialized optical interrogation techniques. Being only a fraction of a millimeter in diameter, they can be directly incorporated into wearables with minimal impact on patient comfort. It has been shown that various optical fiber sensing schemes are sensitive enough to measure vital signs and patient movement. For example, direct intensity transmission measurements through coiled optical fiber mediated by bend loss have been demonstrated as sufficiently sensitive as a wearable sensor to measure heart rate and respiration rate.^24^^–^^26^ Such a sensor has the benefit of being cheap and simple to interrogate but is only a single-point measurement and requires specific packaging to implement the microbending effect. An alternative method is to use fiber Bragg gratings, which can be multiplexed to yield spatial information, and have also been demonstrated for measuring heart rate and respiration rate as a wearable sensor.^25^^,^^27^ However, fiber Bragg gratings are wavelength division multiplexed, which requires expensive optical interrogation equipment. A promising direction of research is the use of interference effects in multimode optical fiber (MMF), i.e., monitoring the speckle output of an MMF.^28^ Being based on optical interference, the speckle output is highly sensitive to perturbations along with the fiber but can be cheaply interrogated using a digital camera. This method has been demonstrated for applications such as force myography^29^ and the measurement of heart rate and respiration rate when attached to a bed.^30^^,^^31^ The complex and sensitive information provided by the specklegram output of an MMF is a topic of recent interest, particularly when combined with machine learning approaches, where it has been shown that spatial information can be extracted without requiring time or frequency domain interrogation schemes^32^^,^^33^ and can be used to extract measurement signals well below noise levels.^34^ While it is possible to extract spatial (multipoint/distributed) sensing information from MMFs using machine learning, this approach fundamentally relies on mode coupling and requires pretraining, with examples including 3000 samples in the case of strong mode coupling in a ring core fiber^33^ and ten thousand or more samples for conventional MMF.^32^^,^^33^ These approaches have so far only demonstrated the classification of a perturbation location without providing quantitative information on the degree of perturbation.

Here, we propose a cheap and simple-to-implement approach to spatially resolved MMF specklegram sensing for use in a smart bed. Our approach takes advantage of the advancements in modern camera technology and requires only cheap optical fiber cable with no additional optical sensor elements. We use an array of seven MMF cables and bundle them at the input and output such that a single coherent light source and single camera are required for interrogation. We present a proof-of-concept demonstration that the sensor array can detect basic movements and breathing of a person on a mattress and can provide basic spatial information using only a simple correlation algorithm. In the future, we anticipate our method could be expanded both in sensitivity and spatial resolution without increasing interrogation costs through further multiplexing and taking advantage of emerging machine learning algorithms.

## 2 Concept

The propagation of coherent light through an MMF produces a speckle output due to the superposition of multiple guided orthogonal modes. For a step-index optical fiber with a circular core the number of modes, N, can be estimated using $N=V^{2}/2$, where V=2πa(NA)/λ is the normalized frequency, a is the core radius, λ is the free space wavelength of the propagating light, and NA is the numerical aperture of the fiber.

The MMF used in this work (OM1 specification) has a core diameter of 62.5  μm, a numerical aperture of 0.275, and we operate at a wavelength of 633 nm. This yields a normalized frequency value of 85.3 and thus has an estimated number of modes of 3640. In practice, less modes may be launched in the fiber if the launching optics have a lower numerical aperture. Mode-dependent loss will also cause some of the highest order modes to be lost, depending on the multimode fiber length and any bending applied. However, the estimated number of modes gives an upper bound estimate that is useful for predicting the complexity of the speckle pattern that will be produced from the multimode fiber.

Each mode propagates with a different phase velocity, which can be represented by an effective refractive index, and associated with each mode is a propagation invariant electromagnetic transverse field profile. As the phase velocity of each mode is different, and each mode profile is different, a complex interference intensity pattern, I, will be formed at the fiber output as described as

**Fig. 1:**
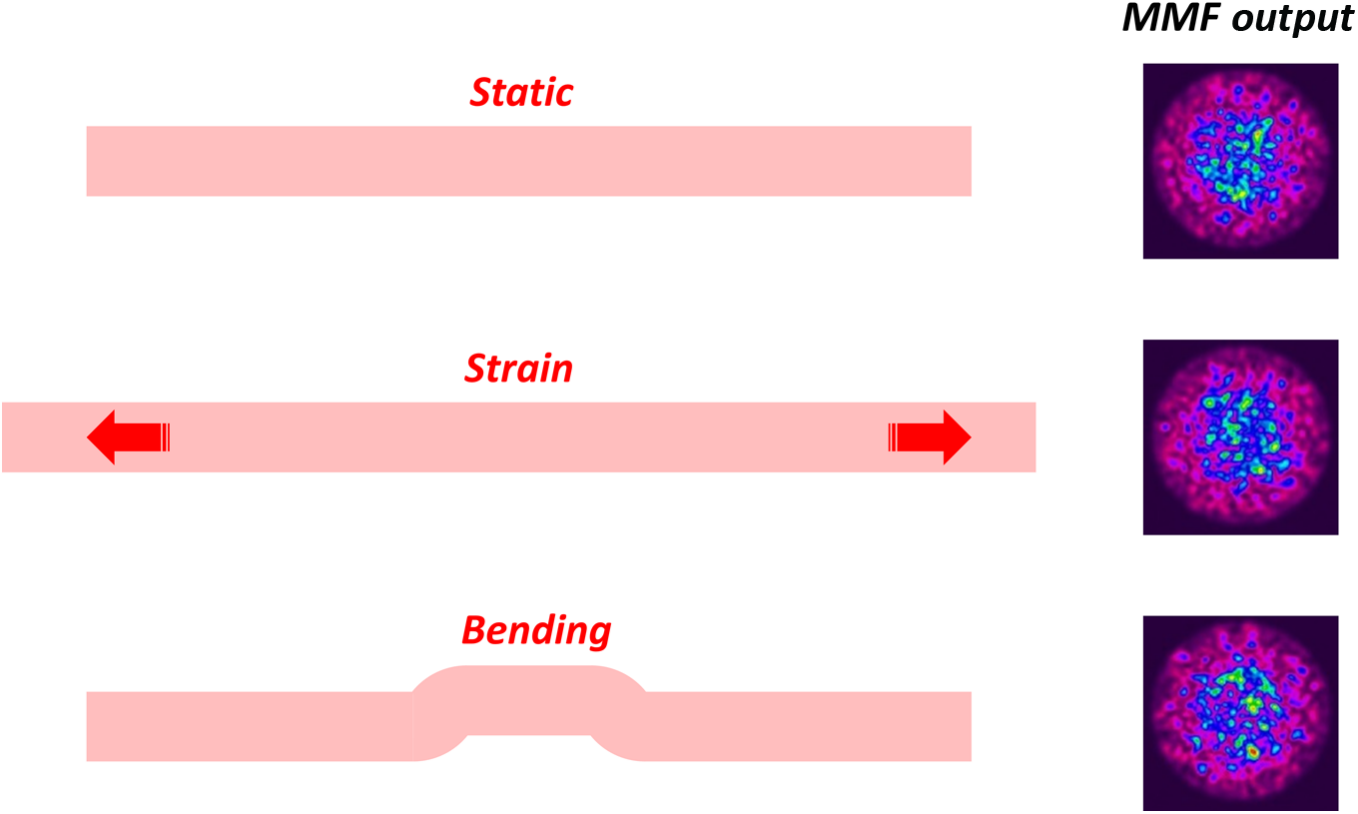
Concept diagram showing how physical perturbations on the MMF, such as strain and bending, can lead to complex changes in the speckle output.

I(x,y)=|∑jaje→^(x,y)exp(i2πλnjeffL)|2, (1)

where $a_{j}$, $n_{j}^{\mathrm{eff}}$, and e→^(x,y) are the mode amplitude, effective refractive index, and normalized electric field distribution of the j’th mode, respectively, and L is the physical length of the fiber. Here, we define the propagation axis of the fiber to be along the z coordinate and the transverse plane is given by the x and y coordinates. Any physical perturbations that occur on the optical fiber, such as strain and bending, will cause a change in the optical path length (the effective index multiplied by the physical length) of the modes. As this is carried by the phase term in Eq. (1), it leads to complex and sensitive changes to the speckle pattern output as schematically shown in Fig. 1.

The next step is to quantify how much the speckle pattern has changed when a physical perturbation has occurred. Many advanced techniques have been used for image transmission through an MMF, such as transmission matrix^35^^,^^36^ and deep learning^37^^,^^38^ approaches. In this work, we use a simpler algorithm based on the dynamic temporal change of a zero normalized cross correlation (ZNCC),^39^ which is given as

$${\mathrm{ZNCC}}_{i}=1-(\frac{1}{\left(t_{i}-t_{i-1}\right)})\frac{\overset{N}{\underset{n=1}{\sum }}(I_{i}(n)-{\overset{¯}{I}}_{i})(I_{i-1}(n)-{\overset{¯}{I}}_{i-1})}{\sqrt{\overset{N}{\underset{n=1}{\sum }}{\left(I_{i}(n)-{\overset{¯}{I}}_{i}\right)}^{2}\overset{N}{\underset{n=1}{\sum }}{\left(I_{i-1}(n)-{\overset{¯}{I}}_{i-1}\right)}^{2}}},$$ (2)

where $I_{i}$ is the camera intensity of the i’th frame of the imaged speckle pattern, $t_{i}$ is the time stamp of the i’th frame, ${\overset{¯}{I}}_{i}$ is the average intensity of the i’th speckle image, and n is the index of the camera image pixels (two-dimensional) with a total number of pixels being N. The ZNCC will range from zero to unity, where zero indicates the speckle pattern of a particular MMF output has not changed since the previous frame while unity shows complete decorrelation from the previous frame. Note that here we consider the frame-to-frame change in ZNCC such that only the dynamic changes are captured, avoiding issues of limited dynamic range and drift that can plague measurements on slow-moving parameters, such as temperature.^39^

We now show in the following sections how this concept can be used to create a simple sensor array by monitoring multiple MMFs simultaneously. Importantly, a single camera and single light source can be used for multiple MMFs so that the spatial information can be scaled without requiring additional detection optics.

## 3 Materials and Methods

### 3.1 Multimode Fiber Bundle

**Fig. 2:**
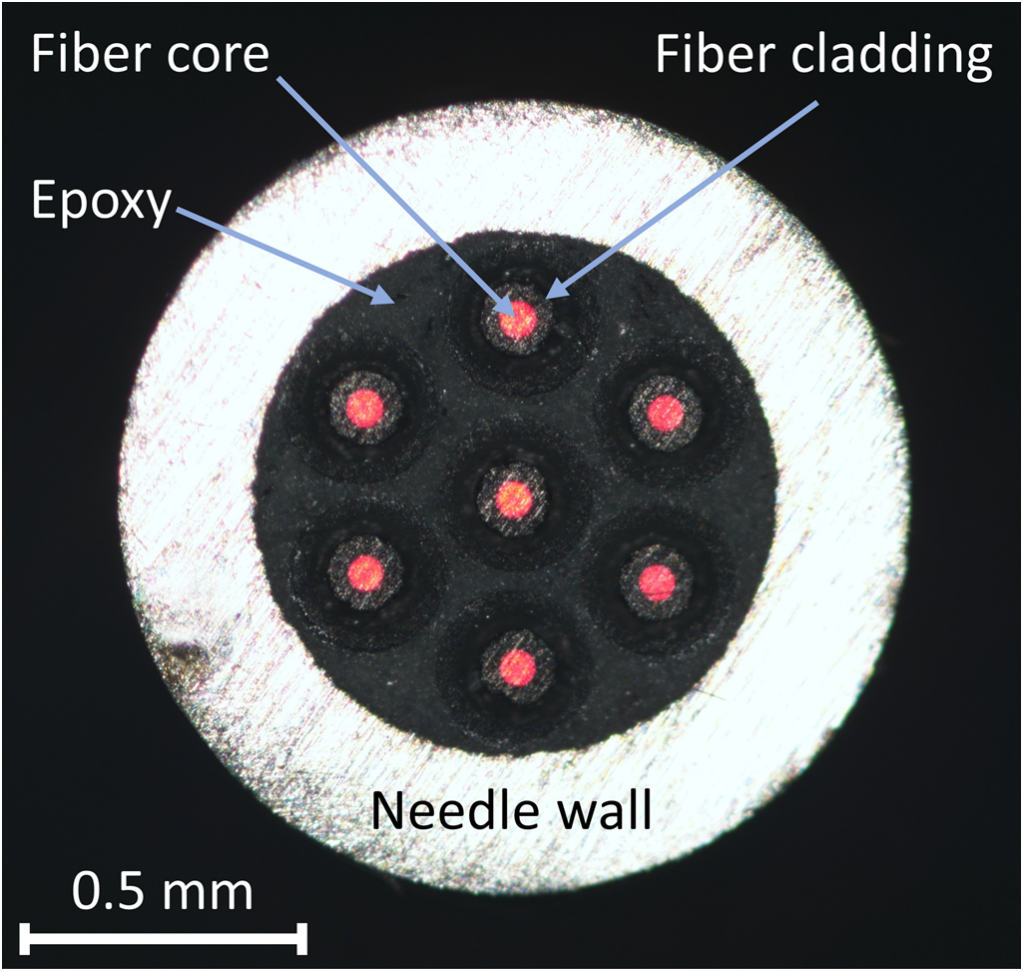
Microscope image of the seven MMFs epoxied into the drawing up needle. The far end of the fibers was illuminated with a red laser source so that the fiber cores were visible under the microscope.

We used seven commercially available and low-cost MMFs with OM1 specification (FS.COM). Each MMF was ∼5-m long, with a core diameter of 62.5  μm, cladding diameter of 125  μm, coating diameter of 250  μm, and outer buffer diameter of 900  μm. The outer buffer was stripped for a length of ∼300  mm, bundled into an 18-gauge drawing-up needle, and fixed into place using epoxy. The tight-fitting of the coated fiber meant that the seven fibers naturally settled into a hexagonal arrangement. After the epoxy had cured, the tip of the fibers protruding from the needle were cut and polished to a flat surface, as shown in Fig. 2. This process was repeated for the far end of the fibers for both optical launching and imaging.

### 3.2 Optical Detection

**Fig. 3:**
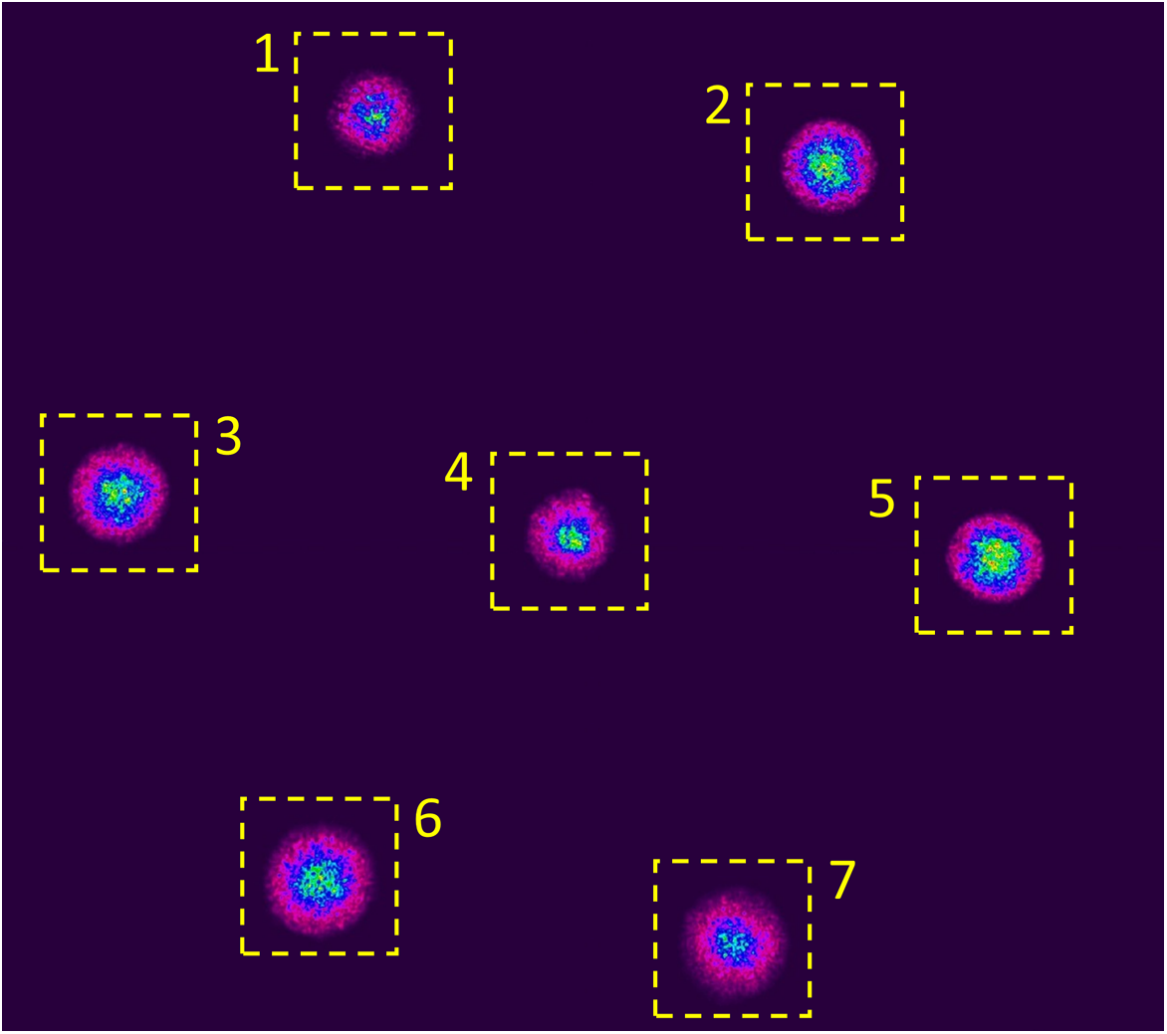
Example camera image of the output from the multimode fiber bundle. Yellow-dashed boxes show the 200×200  pixel regions used for the ZNCC calculations for each fiber.

The bundled fiber ends were mounted onto three-axis translation stages for positioning of the launching laser beam and imaging onto a camera. Light from a coherent visible wavelength (633 nm) light source was coupled with a single-mode fiber (Thorlabs, 630HP) and then collimated using a 10× microscope objective to yield a beam with ∼3.4-mm diameter. The collimated beam was then directed onto the end face of the MMF bundle, which was intentionally overfilled to have relatively uniform excitation across the seven fibers. The far end of the fiber bundle was then imaged onto a CCD camera (Ophir-Spiricon, SP300, 12-bit, grayscale) using a 10× microscope objective. The full camera resolution was used (1928×1448  pixels) at a frame rate of ∼2  Hz. An example of the image is shown in Fig. 3, where the dashed boxes show the 200×200  pixel regions used for analysis (ZNCC) for each individual MMF speckle pattern.

### 3.3 Sensor Attachment to a Mattress

**Fig. 4:**
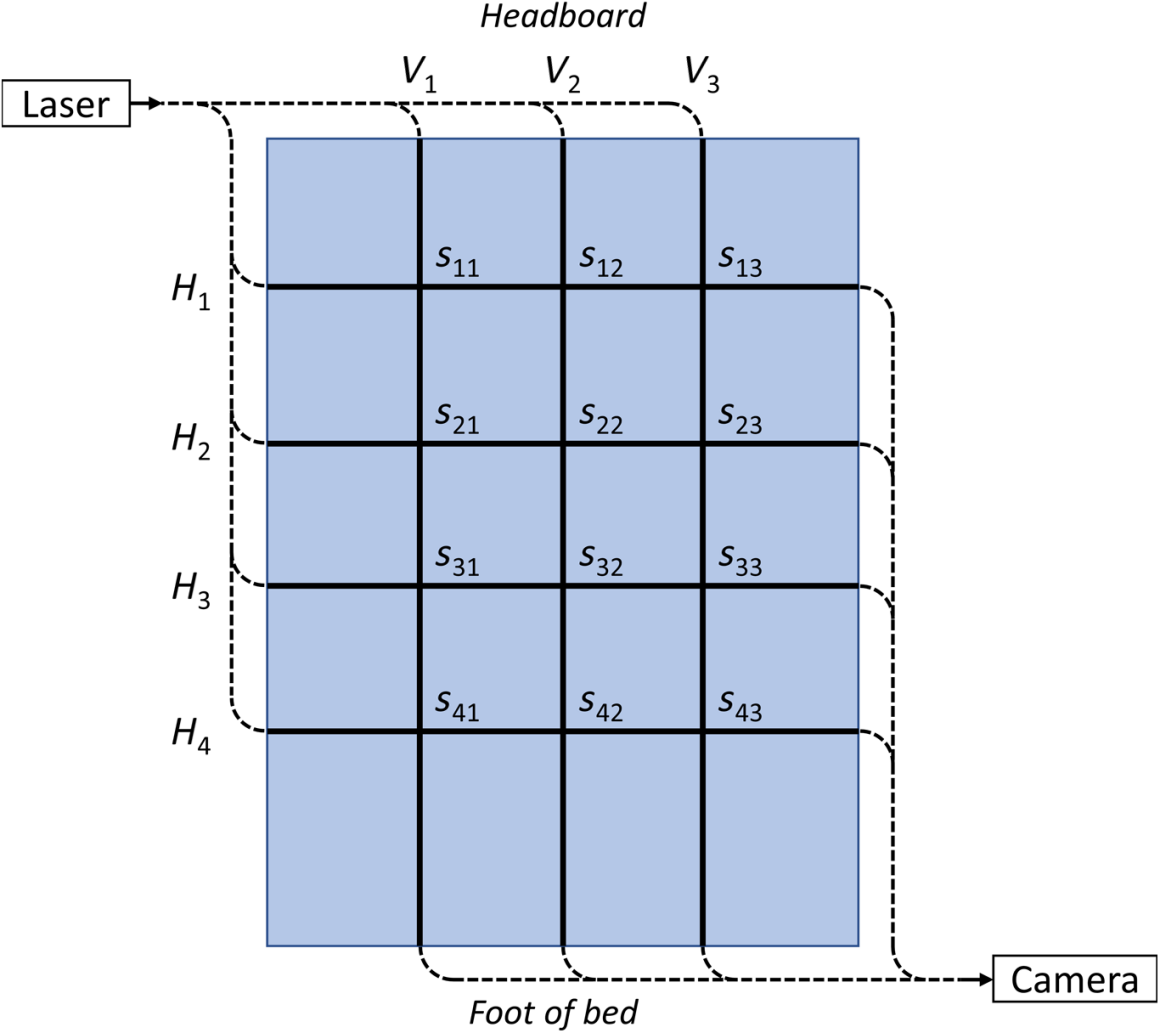
Schematic diagram of the multimode fiber array. The seven MMFs were bundled at the input and output for launching the laser light and monitoring the output on a camera, respectively. The three vertical ($V_{i}$) lines and four horizontal ($H_{j}$) lines indicate the location of the seven multimode fibers. There are twelve corresponding intersection points, $s_{ij}$.

The seven multimode fibers were fixed to a spring mattress with a foam top. Three fibers were laid vertically along the bed while four were laid horizontally, leading to 12 crossing points as shown schematically in Fig. 4.

As will be shown experimentally in Sec. 4, multiplying the sensor response of each horizontal and vertical pair of MMFs yields spatial information on the movement that occurs at these crossing points. In principle, mathematically, we have 12 unknowns (crossing points, $s_{ij}$) for seven knowns (ZNCC for seven MMFs), making it impossible to determine a unique value for the movement that has occurred at each crossing point. However, in practice, the movement often occurs in clustered locations on the mattress where several crossing points show minimal signal. Therefore, in this work, we use a simple algorithm where the vertical and horizontal signals are multiplied to yield basic spatial information on movement as given as

$$s_{ij}={\mathrm{ZNCC}}_{i}\times {\mathrm{ZNCC}}_{j},$$ (3)

where ${\mathrm{ZNCC}}_{i}$ and ${\mathrm{ZNCC}}_{j}$ are the ZNCC scores for the three vertical MMFs and the four horizontal MMFs, respectively.

**Fig. 5:**
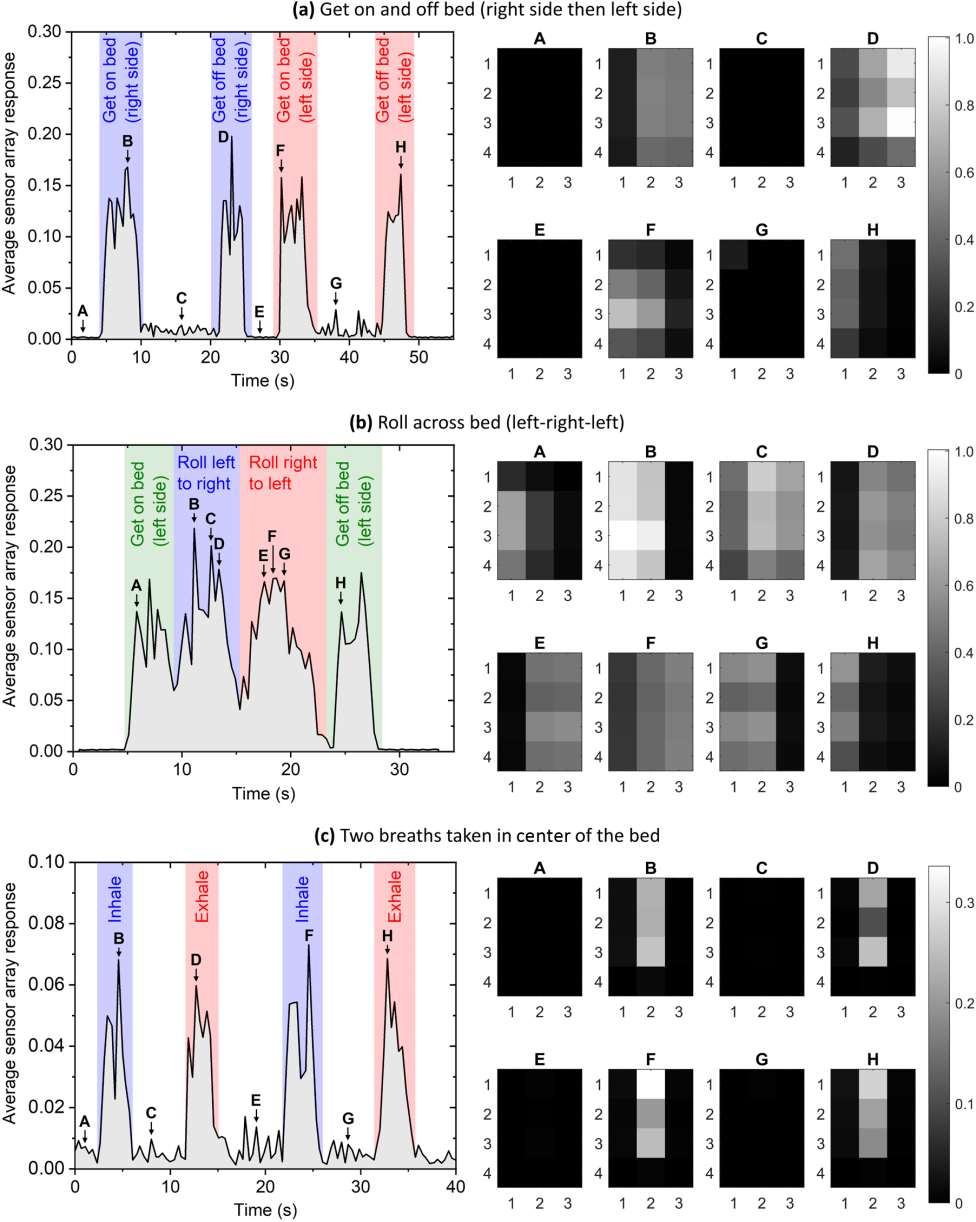
Multimode fiber sensor array test results. Left-hand side plots show the ZNCC score averaged across the seven MMFs. The right-hand images show the scores from Eq. (3) for the twelve intersection points. (a) Participant got on and off the bed, first the right-hand side then the left-hand side, corresponding to Fig. 4 (Video 1, MP4, 5.6 MB [URL: https://doi.org/10.1117/1.JBO.27.6.067002.1]). (b) Participant rolled across the bed, first from left to right, then right to left (Video 2, MP4, 3.9 MB [URL: https://doi.org/10.1117/1.JBO.27.6.067002.2]). (c) Participant laid on the center of the bed without moving and took two breaths (Video 3, MP4, 3.3 MB [URL: https://doi.org/10.1117/1.JBO.27.6.067002.3]). Videos also display the array outputs using the linear programming approach, which shows qualitatively similar results. Further information on the linear programming approach is given in the Supplementary Material.

We compare this to a linear-programming (LP)-based approach to solve for crossing points, $S_{ij}$, using the output ZNCC from the seven MMFs. This approach, although less intuitive, is both mathematically rigorous and computationally tractable, except in certain extreme cases. The objective is to determine the unknown matrix of positive entries, $S_{ij}$, from the output ZNCC values for various horizontal $\left\{H_{i}\right\}$ and vertical $\left\{V_{j}\right\}$ fibers. It is assumed that the ZNCC value of a particular fiber is a sum of all the crossing points along with that fiber, $H_{i}=\sum_{j}S_{ij}$ and $V_{j}=\sum_{i}S_{ij}$. These equations impose linear constraints on the entries of the matrix, $S_{ij}$. A feasible matrix of all positive entries, which satisfies these constraints is obtained numerically using the built-in *linprog* function in MATLAB. A detailed mathematical derivation of the approach and discussion of its validity is provided in the Supplementary Material. The results of the LP approach match closely with the results of more intuitive but approximate, multiplication approach discussed above (see Videos 1–3, listed in the caption to Fig. 5). A discussion of when the two approaches will give the same answer, and relative merits and demerits of each when they differ, is also provided in the Supplementary Material.

## 4 Results and Discussion

We have performed a proof-of-concept demonstration of the multimode fiber sensor array for smart bed monitoring applications. Three procedures were performed to demonstrate potential movements of interest: (a) getting on and off the mattress, (b) rolling across the mattress, and (c) breathing. This demonstration was performed by a healthy adult (male, 35 years old, 82 kg) with the results shown in Fig. 5. The left-hand side traces are the ZNCC score averaged across the seven multimode fibers, indicating total movement but without spatial information. The right-hand side figures show spatial information from the crossing points, $s_{ij}$. The spatial plots in Figs. 5(a) and 5(b) show that movement across the left and right side of the mattress can be reasonably discriminated. Figure 5(c) shows the response when the participant did not move but laid flat at the center of the mattress and took two breaths. The breaths in and out were intentionally separated in time to observe the response of the sensor array. The sensor array is sufficiently sensitive to detect breathing, while the location of the participant could also be identified. As expected, the strength of the signal from breathing is less than the gross movement in Figs. 5(a) and 5(b).

Careful analysis of Fig. 5(a) also shows that the sensor array successfully detects the presence of a person on the mattress in the absence of movement. This is to be expected due to movements associated with breathing and heartbeat. That is, in region A (off the bed) the ZNCC value with an uncertainty given as three standard deviations is 0.0019±0.0013 while in region C (on the bed) this value is 0.0091±0.0037, showing that these two states are clearly distinguishable. Therefore, the MMF sensor array could be used as an alternative to hospital bed pressure sensors for detecting when a patient leaves a bed.

**Fig. 6:**
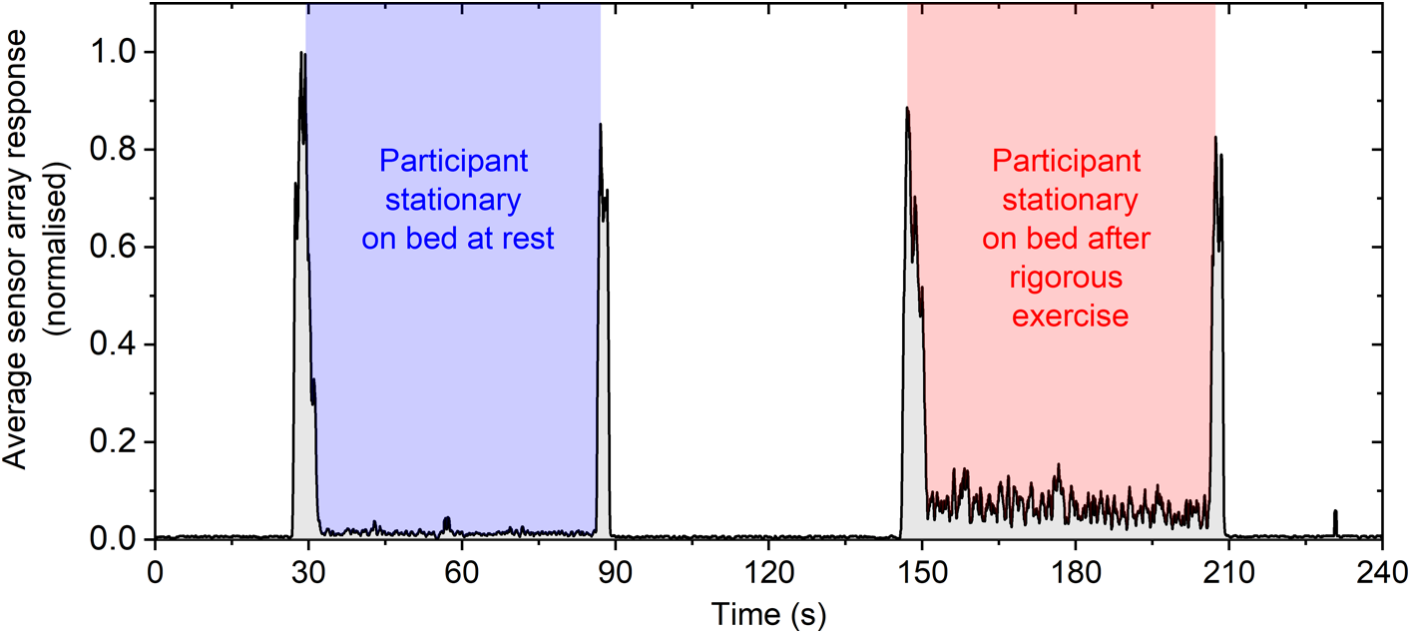
Multimode fiber sensor array test average response where the participant lay stationary on the mattress, before and after a 60-s interval of rigorous exercise.

A further test was performed to demonstrate the ability of the sensor array to detect the presence of a person on the mattress due to their physiological state. In this test the participant lay stationary on the mattress for 60 s after a period of inactivity, that is, at rest. The participant then undertook 60 s of rigorous exercise (running) and then returned to the mattress motionless for 60 s. The average sensor response over this test is shown in Fig. 6. We note that the trace was normalized, as the optical fiber imaging alignment had been repositioned from the results shown in Fig. 5 (experiment performed on different days) and thus the magnitude of the sensor response is not directly comparable. The results clearly show that the sensor array responds strongly to the physiological effects of the rigorous exercise, primarily increased heartrate and breathing. While the acquisition rate used during these tests (2 Hz) was not sufficient to resolve the heart and respiration rates in these scenarios, the overall increased response of the sensor array clearly shows the ability to detect this level of motion.

In addition to temporal resolution considerations, further development of the sensor array approach could be to increase the spatial resolution using additional MMFs. In this work, the use of seven optical fibers meant that they could be bundled in a natural hexagonal arrangement for simple connector fabrication. This allowed for a 3×4 sensor configuration, which was a compromise between the ease of fabrication while obtaining basic spatial information. Future arrays could be fabricated with, e.g., 19 MMFs, which can also form a hexagonal bundle. This could allow, e.g., 9×10 arrays with 90 crossing points.

It is envisaged that this MMF sensor array concept could allow for noninvasive monitoring of patients in hospital and nursing home settings, where the overarching aim is for improved health outcomes and reducing the human resources burden of manual monitoring of patients. We speculate healthcare tasks that may particularly benefit include:

### 4.1 Falls Risk Reduction

Falls by in-hospital patients are a significant healthcare cost and must be prevented.^40^ Existing weight-based hospital bed pressure sensors are limited in that they generally cannot predict when a patient will exit the bed, until part of the patient is already touching the floor, leaving little time for nursing staff to respond and assist the mobilizing patient. The complex spatial information of the MMF array may provide early warning for at-risk patients.

### 4.2 Pressure Area Care

Pressure injuries acquired in hospital and care settings continue to be a significant burden on the health system and reduce the quality of life for patients, despite being generally preventable. ^41^^,^^42^ Key components of prevention include adequate movement, and altering the magnitude and/or duration of pressure loading such as through regular patient turns.^43^^,^^44^ Spatiotemporal quantification of a patient’s history of movement would allow for better-informed nursing decisions to prevent pressure injuries. For example, alerting nurses that a patient requires pressure area care if the patient has not made enough pressure-related adjustments in their bed in recent hours.

### 4.3 Monitoring of Respiration Rate and Work of Breathing

Respiration rate is often considered the most critical vital sign for nursing staff to monitor accurately for early detection of patient deterioration.^45^^–^^47^ This is either done manually by counting over 1 min, or, continuously using monitors that measure either electrical activity in the chest, or carbon dioxide from the breath. Manual methods are often completed poorly or in haste by nurses.^45^ Both continuous methods require devices to be attached to the patient, either to the chest, as a mask on the face, or a ventilator; neither of which may be appropriate in ambulatory patients and/or in acute wards, or aged care settings. The ability to continuously, noninvasively, and cheaply monitor respiration rate would allow far greater prevalence of monitoring this important vital sign. Furthermore, the ability to measure the work of breathing, which is the energy required per breath and the relative use of accessory muscles, is another significant observation that is indicative of respiratory distress yet is difficult to quantify outside of clinician observation.^48^ The spatial information of the MMF array may allow noninvasive quantification and monitoring of changes to the work of breathing.

## 5 Conclusions

We have presented a proof-of-concept demonstration, for the first time to our knowledge, a cheap and scalable smart bed motion detection scheme using an array of MMFs with a single laser source and single camera. This provides both information on the magnitude of the movement of a person on the smart bed and spatial information of where that movement has occurred. The sensor array is also sufficiently sensitive to monitor breathing and more generally the presence of a person stationary on the bed. While for this proof-of-concept demonstration we have only shown the detection of a few basic movements, in the future, the scheme can be used as a platform for high-spatial-density sensing and more advanced signal analysis, such as machine learning to capture and analyze more complex patient movements. Future research opportunities include increasing the spatial and temporal resolution, designing methods for best integrating the sensor array into the mattress, and verification for clinically relevant patient movements.

## Code, Data, and Materials Availability

Data underlying the results presented in this paper may be obtained from the authors upon reasonable request.
