# Supplementary material for: Multimode optical fiber specklegram smart bed sensor array
Source: J Biomed Opt. 2022 Jun 24;27(6):067002. doi: 10.1117/1.JBO.27.6.067002 (PMC9231555; doi:10.1117/1.JBO.27.6.067002)
Supplement: Supplementary file 4 [file JBO_027_067002_SD004.pdf]

Multimode optical fiber specklegram smart bed sensor array: supplementary material

Stephen C. Warren-Smith,[a,b,c,*] Adam D. Kilpatrick,[d,e] Kabish Wisal,[f] and Linh V. Nguyen[a,b]
[a]Future Industries Institute, University of South Australia, Mawson Lakes, SA 5095, Australia
[b]School of Physical Sciences and the Institute for Photonics and Advanced Sensing, The University of Adelaide, Adelaide, SA 5005, Australia
[c]Australian Research Council Centre of Excellence for Nanoscale Biophotonics, The University of Adelaide, Adelaide, SA 5005, Australia
[d]Adelaide Nursing School, Faculty of Health and Medical Sciences, The University of Adelaide, Adelaide, SA 5005, Australia
[e]Royal Adelaide Hospital, Adelaide, SA 5000, Australia
[f]Department of Physics, Yale University, New Haven, CT 06520, USA

1. LINEAR PROGRAMMING APPROACH DERIVATION

We showed in our paper that a horizontal and vertical array of optical fibers, as shown in Fig. S1, can be used to measure spatially varying movement.

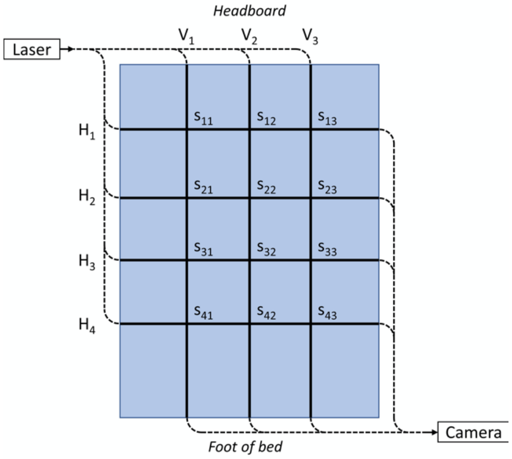

Fig. S1. Setup for measuring spatially varying sensory activity using MMFs.

We measured the ZNCC values of various horizontal and vertical fibers, denoted by $\{H_i\}$ and $\{V_j\}$, respectively. Since we cannot directly access the matrix encoding the spatial information for the sensory activity, S_{ij}, it needs to be reconstructed from various fiber ZNCC values. Fortunately, reconstructing S_{ij} is a feasible task since the ZNCC value for a particular fiber must be a net result of the sensory activity along that fiber. Here, we present a mathematical framework of a linear programming based approach to reconstruct S_{ij}. Let the number of horizontal and vertical fibers be m and n, respectively. The ZNCC value for a particular fiber is a sum of all the sensory activity along that fiber:

$$H_i = \sum_{j=1}^{n} S_{ij}\,, \;\; \forall i \in \{1,2,..m\}, \tag{S1}$$

$$V_j = \sum_{i=1}^{m} S_{ij}\,, \;\; \forall i \in \{1,2,..n\}. \tag{S2}$$

In addition, sensory activity at each crossing needs to be non-negative:

$$S_{ij} \geq 0, \;\; \forall i \in \{1,2,..m\}, \;\; j \in \{1,2,..n\}. \tag{S3}$$

We have $m * n$ unknown quantities, S_{ij}, $m + n$ linear, equality constraints, and $m * n$, linear, inequality constraints. The restriction of unknown quantities, S_{ij}, to only positive values renders the usual matrix inversion techniques to solve linear equations inviable. However, all the constraints involved here are linear, thus constrained linear optimization approaches such as Linear Programming (LP) would be readily applicable. It should be noted that since the number of equality constraints $(m + n)$ are less than the number of unknowns, $m * n$ for any $m, n > 2$, the solution we obtain will be non-unique. However, this approach allows us to obtain a feasible solution, which respects all the constraints imposed by the output ZNCC values and therefore captures essential features of the sensory activity. To convert the inverse problem into a linear optimization problem we introduce $m + n$ error variables, $\{e_i^h\}$ for m horizontal fibers and $\{e_j^v\}$ for n vertical fibers. These error variables keep track of deviation from each individual constraint given in Eqs. S1 and S2:

$$\sum_{j=1}^{n} S_{ij} - e_i^h = H_i \, , \forall \ i \in \{1, 2, ..m\}, \tag{S4}$$

$$\sum_{i=1}^{m} S_{ij} - e_j^v = V_j \, , \forall \ j \in \{1, 2, ..n\}. \tag{S5}$$

Next, we define our objective function to be minimized as the sum of all the error variables:

$$F = \sum_{i=1}^{m} e_i^h + \sum_{j=i}^{n} e_j^v. \tag{S6}$$

The error variables are restricted to be non-negative quantities so that individual errors are minimized, instead of cancellation, upon optimization. The constraints in Eqs. S3, S4 and S5 together with the objective function in Eq. S6 forms a standard linear programming system. Its solution can be obtained quickly and efficiently by using the built-in function, *linprog*, in MATLAB. In the case when the minimum value of the objective function is zero, all the error variables must be individually zero, since they are non-negative, which ensures that all the constraints in Eqs. S1 and S2 are satisfied. Thus, the resulting solution, S_{ij}, is a feasible sensory activity matrix which incorporates all the known information exactly.

2. COMPARISON TO MULTIPLICATION BASED APPROACH

In our paper, we used another simple and intuitive, albeit approximate, method to recover the sensory activity matrix. In this method, a particular element of sensory activity matrix, S_{ij} is obtained as a multiplication of ZNCC values in the ith horizontal and j^{th} vertical fiber, $S_{ij} = H_i V_j$. We show that, in the videos attached for different activities such as breathing, rolling on the bed etc, results of both the multiplication based approach and LP approach track each other closely and are reasonably able to capture the activity being tested. The relatively high performance of both the methods is a result of the presence of a-priori structure within the sensory matrix due to additional constraints such as localisation and continuity of test subjects. We would like to point out, since we are trying to recover more unknowns $(m * n)$ than the number of constraints $(m + n)$, any solution whether based on the multiplication approach or LP approach will be approximate. Therefore, success of both the approaches depends on the prior structure of the sensory matrix. We provide a few examples of typical structures in the sensory matrix and discuss relative performance of both the LP approach and the multiplication based approach. In particular, we discuss three extreme cases, illustrating the conditions under which each approach works best or fails the most. We start by having a true S_{ij} matrix with a particular structure and generate individual fiber ZNCC values, $\{H_i\}$ and $\{V_j\}$, by summing across rows and columns respectively. Next, we use these fiber ZNCC values as inputs and reconstruct the sensory matrix, S_{ij}, using both multiplication and LP approaches and compare the results to the true matrix. For demonstration purposes, we have chosen $m = 4$ and $n = 3$ as these are the numbers also used in our experimental studies.

A. Single Non-Zero column

One of the relevant structures in the sensory activity matrix is a presence of a single non-zero column and the remaining entries being zero. In our experiments, this would be the case when a person lies vertically in a particular section (left, right or middle) of the bed. The results for recovering such a matrix is given in Fig. S2. It can be seen that both the LP approach and

multiplication approach reproduce the original matrix exactly. This is the case since the number of relevant non-zero unknowns (m) are not greater than number of constraints ($m + n$). Fortunately, this is the case the in many physically relevant situations even when the matrix is not quite a single non-zero column.

B. Full Matrix

The LP based approach works best when the number of non-zero elements in the matrix are not greater than the number of constraints. Therefore, the case in which this approach performs worst is in when the actual matrix is a completely full matrix. The results for such a case are presented in Fig. S3. The LP approach produces a matrix having just two non-zero diagonals instead of a full matrix. This happens because an appropriately scaled diagonal matrix produces the same sums of rows and columns as a full matrix. Interestingly, the multiplication approach still works well in this case. This is because the multiplication approach has an averaging effect which does not change the result if the original matrix is completely full.

C. Diagonal Matrix

The multiplication based approach to recover S_{ij} ends up incorporating effects of other entries across the same row and column (cross talk). This leads to averaging of the actual matrix across rows and columns with non zero sums. The case in which this effect produces most errors is when all the non-zero entries are present on a diagonal of the original matrix. The results for this case are shown in Fig. S4. The multiplication approach produces a matrix which is completely full, except for the last row which is completely empty. As expected, the LP approach works well in this case, since there are only a few non-zero elements.

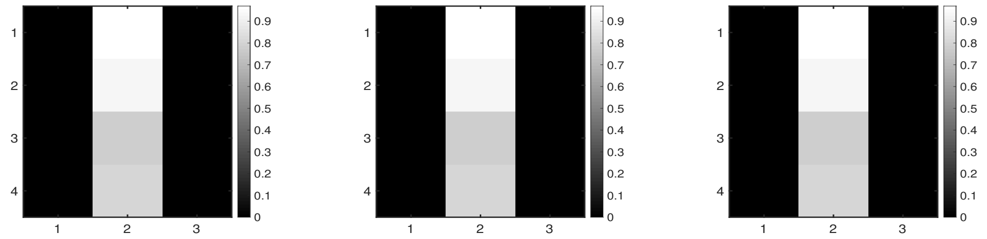

(a) Actual **(b)** LP Approach **(c)** Multiplication approach

Fig. S2. Reconstruction of sensory activity matrix with a single non-zero column.

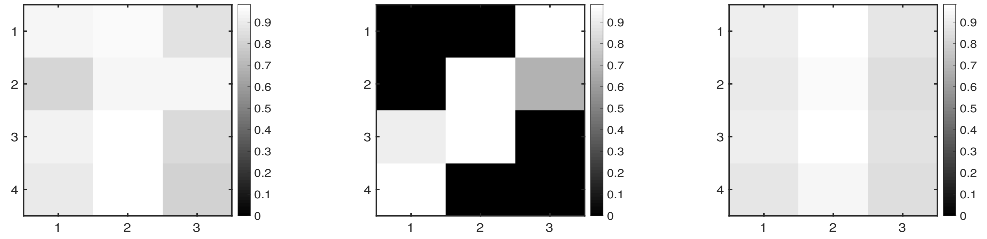

(a) Actual **(b)** LP Approach **(c)** Multiplication approach

Fig. S3. Reconstruction of a completely full sensory activity matrix.

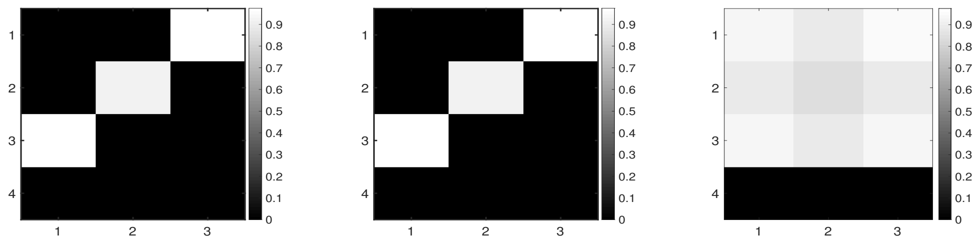

(a) Actual **(b)** LP Approach **(c)** Multiplication approach

Fig. S4. Reconstruction of sensory activity matrix with only a non-zero diagonal.
